# Comparative analysis of microbial diversity and clinical outcomes in critically ill patients with and without malignancies: a single-center retrospective cohort study

**DOI:** 10.3389/fmicb.2026.1777861

**Published:** 2026-03-03

**Authors:** Jinwei Yao, Fangzhou Wang, Haichao Li, Ruoxi Zhang, Guofeng Ji, Dachuan Liu

**Affiliations:** Department of General Surgery, Xuanwu Hospital, Capital Medical University, Beijing, China

**Keywords:** antimicrobial resistance genes, cancer, metagenomics, patient prognosis, septic shock

## Abstract

**Background:**

Sepsis and septic shock are severe complications for surgical malignancy patients. Conventional diagnostics often fail to capture the complex infectome in these populations. This study aimed to characterize the distinct microbial and resistome landscapes in cancer versus non-cancer patients using multi-site metagenomic next-generation sequencing (mNGS) to support specific antimicrobial strategies.

**Methods:**

We conducted a single-center retrospective cohort study at the General Surgery ICU of Xuanwu Hospital, including 107 septic shock patients (52 cancer; 55 non-cancer). mNGS was performed on blood, bile, ascitic fluid, and bronchoalveolar lavage samples to identify pathogens and antibiotic resistance genes (ARGs). Findings were analyzed for their association with ICU length of stay and mortality.

**Results:**

Cancer patients were significantly older (median 68 vs. 51 years, *p* < 0.0001) with higher comorbidity scores (CCI: 7.0 vs. 4.0, *p* = 0.006). However, mNGS revealed a lower pathogen detection rate in cancer patients (53.85% vs. 85.45%, *p* = 0.0006) and a lower incidence of bacteremia (25.0% vs. 45.45%, *p* = 0.0426). Cancer patients had shorter ICU LOS (9 vs. 13 days, *p* = 0.0369) and antibiotic durations (7 vs. 11 days, *p* = 0.0368). Dominant pathogens included *Klebsiella pneumoniae* and *Enterococcus faecium*, harboring diverse ARGs across beta-lactam and aminoglycoside categories. Multivariate Cox regression identified IL-6 (*p* = 0.018) was significant prognostic indicators for cancer patients. We also examined the distribution of virulence factors, despite their low detection rates.

**Conclusion:**

Septic shock in cancer patients exhibits a unique resistome signature and distinct prognostic drivers. The identification of microbial targets via mNGS was associated with the implementation of targeted antimicrobial strategies and inflammation monitoring. These findings suggest that mNGS provides valuable molecular insights that may support clinical management and prognostic stratification for cancer patients in the surgical ICU.

## Introduction

Sepsis has long been a global health challenge, characterized by life-threatening organ dysfunction caused by a dysregulated host immune response to infection (Singer et al., 2016). In the intensive care unit (ICU), sepsis is a leading cause of mortality, particularly when it progresses to septic shock, where metabolic and circulatory abnormalities significantly increase the risk of death (Evans et al., 2021). Recent epidemiological data indicate that over 20% of sepsis-related hospitalizations involve patients with underlying malignancies (Rhee et al., 2017). These patients are uniquely vulnerable due to immune impairment caused by the malignancy itself or secondary to cytotoxic therapies, surgery, and long-term use of invasive devices (Tellapragada et al., 2020). The clinical course of sepsis diverges significantly between patients with and without cancer. Compared to non-cancer populations, cancer-related sepsis is associated with higher in-hospital mortality and a higher incidence of opportunistic infections (Cheng et al., 2025). Despite the clinical recognition of these risks, traditional culture-based diagnostic methods often fail to capture the full landscape of the infectome, including fastidious organisms, dormant pathogens, and the complex distribution of antimicrobial resistance, due to their limited sensitivity and time-consuming nature (Barlam et al., 2016).

The emergence of metagenomic next-generation sequencing (mNGS) has revolutionized the diagnosis and study of infectious diseases in the ICU. Unlike targeted PCR or culture, mNGS provides an unbiased, comprehensive analysis of the microbial environment by sequencing all genetic material in a clinical sample (Liu et al., 2025). In the context of the ICU, mNGS not only improves the pathogen detection rate in sterile sites such as blood and bile but also allows for the profiling of antibiotic resistance genes (ARGs) and virulence factors, which are critical determinants of patient prognosis (Wu et al., 2025; Zhao et al., 2024). However, while mNGS has been increasingly applied to general sepsis, there is a paucity of research utilizing multi-site metagenomic data to specifically compare the microbial signatures between cancer and non-cancer patients. We hypothesized that surgical malignancy patients harbor a distinct resistome profile and specific virulence factor distribution, which independently drive the severity of inflammation and clinical outcomes beyond traditional severity scores.

In this study, we employed mNGS to analyze and compare the microbial composition, drug resistance profiles, and virulence landscapes across multiple anatomical sites in cancer and non-cancer patients admitted to the ICU. By correlating these molecular findings with clinical parameters and long-term prognosis, we aim to provide a deeper understanding of the infectious complexities in the oncology population and offer evidence-based insights for precision treatment in critical care.

## Materials and methods

### Data collection and processing

**Figure 1:**
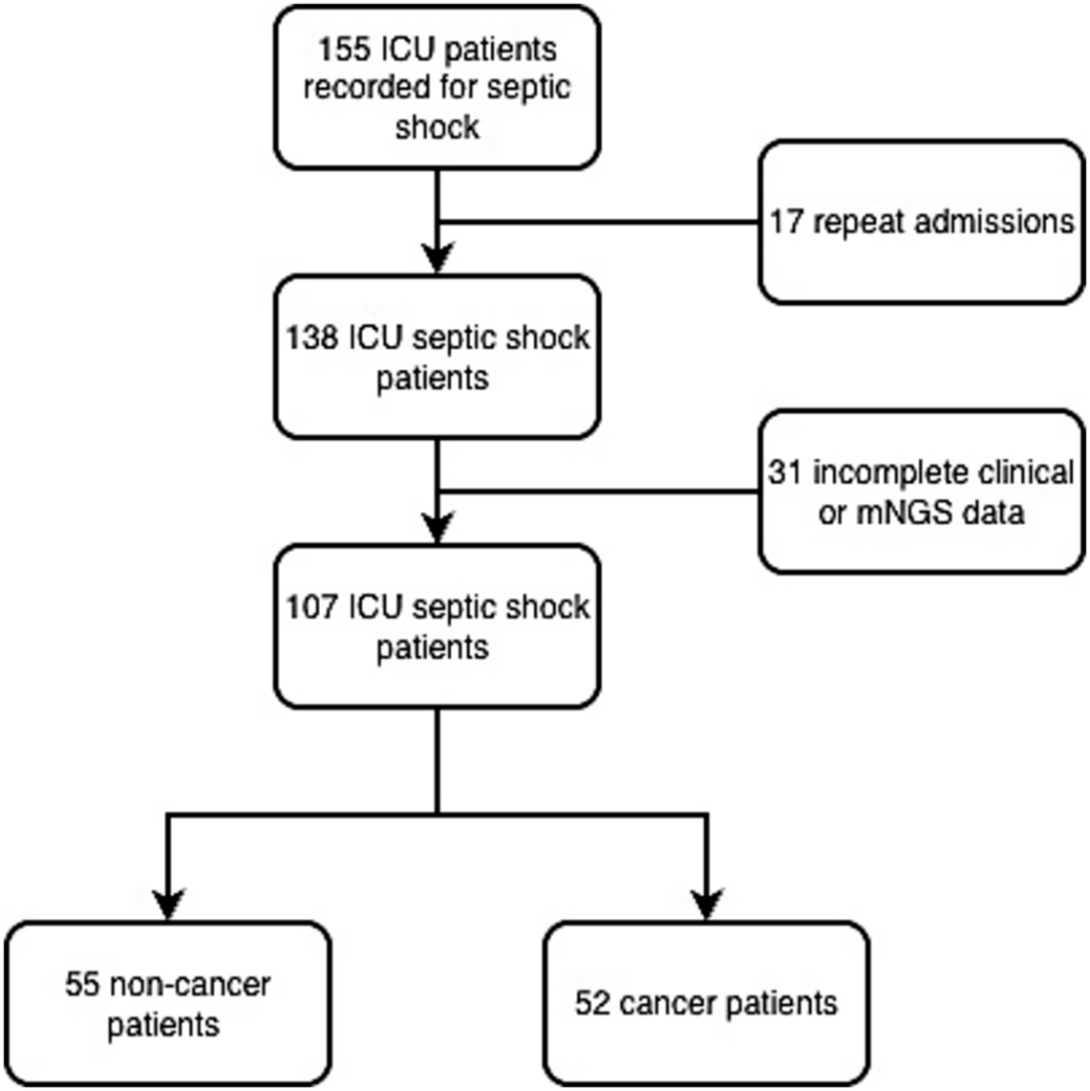
Flow diagram of patient screening and enrollment.

This retrospective, monocentric cohort study was conducted in the general surgery ICU of Xuanwu hospital (January 1, 2023, to November 30, 2025). Inclusion and exclusion criteria specific criteria are detailed in Figure 1 and Supplementary Table S1. Briefly, inclusion required: (1) Age ≥18; (2) Diagnosis of septic shock per sepsis-3 criteria; (3) mNGS testing within 24 h of ICU admission. Exclusion criteria were operationalized as: (1) Pregnancy; (2) ICU stay <24 h; (3) Missing data for primary outcomes or any baseline laboratory variables. Septic shock was defined according to the sepsis-3 criteria and identified via ICD-10 codes. To ensure independence of observations, only the first ICU admission was analyzed for patients with multiple stays. Patients were categorized into two groups: those with underlying malignancies (gastrointestinal and hepatobiliary cancers) and those without. For patients with ICU admissions for septic shock, each stay was analyzed independently. Epidemiological, clinical, and biochemical data were extracted from electronic medical records and standardized, with key parameters spanning demographics (age, sex, weight), comorbidity and health status [assessed using the Charlson Comorbidity Index (CCI) and APACHE II score], therapeutic interventions (including pre-ICU and ICU-based therapies such as antibiotic administration, mechanical ventilation, renal replacement therapy, and transfusion support), and clinical outcomes (including ICU length of stay, ICU mortality, in-hospital mortality). Clinical samples for mNGS (including blood, bile, ascitic fluid, and bronchoalveolar lavage fluid) were consistently collected within 24 h of the onset of septic shock or upon ICU admission. In most cases, sampling was performed immediately following the initial clinical suspicion of infection and prior to any significant modifications to the empirical antimicrobial regimen. Definitions and bias control the cancer status was defined as histologically confirmed gastrointestinal or hepatobiliary malignancy, with details on TNM stage and chemotherapy or immunotherapy status extracted to account for severity. Selection bias was acknowledged due to the surgical specialty of our center. The primary outcome was in-hospital all-cause mortality. Secondary outcomes included ICU length of stay and antibiotic duration. Antimicrobial stewardship was guided by institutional protocols based on IDSA guideline (Barlam et al., 2016), incorporating mNGS results for targeted adjustments.

### Sample collection and mNGS processing

To compare the microbial landscape, clinical samples including peripheral blood, ascitic fluid, bile, and bronchoalveolar lavage fluid (BALF) were collected. In our clinical practice, empirical antimicrobial therapy is initiated immediately upon the clinical suspicion of septic shock. Consequently, mNGS sampling was performed concurrently with or within the first few hours of antibiotic initiation. BALF and other body fluid sample DNA was extracted using the TIANamp Magnetic DNA Kit (Tiangen, Beijing, China) according to the manufacturer’s protocols. Plasma was prepared from blood samples and circulating cell-free DNA was isolated from plasma follow the instructions in QIAamp Circulating Nucleic Acid Kit (Qiagen, Germany). The quantity and quality of DNA was assessed with the Qubit 2.0 (Thermo Fisher Scientific, United States) and NanoDrop 8000 (Thermo Fisher Scientific, United States), respectively. DNA library construction was performed using the Hieff NGS C130P2 OnePot II DNA Library Prep Kit (Yeasen Biotechnology, Shanghai, China), adhering to the manufacturer’s guidelines. Agilent 2100 system was used for quality control of the DNA libraries, and sequencing was carried out on the DIFESEQ-200 platform (Dinfectome, Nanjing, China) employing a 50-base single-end, approach yielding approximately 20 million reads for the metagenomic workflow.

### Bioinformatics analysis

We use in-house developed bioinformatics pipeline for pathogen identification (Lv et al., 2024). Briefly, high-quality sequencing data were generated by removing low quality reads, adapter contamination, duplicated and shot (length <36 bp) reads. Human host sequences were identified by mapping to human reference genome (hs37d5) using bowtie2 software (version 2.2.6). Reads that could not be mapped to the human genome were retained and aligned with microorganism genome database for pathogens identification. Our microorganism genome database contained bacteria, fungi, virus and parasite genomic sequences (download from https://www.ncbi.nlm.nih.gov/).

Subsequently, we applied specific criteria to ascertain positive results from mNGS, as referenced in prior studies (Lv et al., 2024; Xu et al., 2023). The criteria for a positive detection were as follows: (1) At least one species-specific read was required for the detection of Mycobacterium, Nocardia, and *Legionella pneumophila*; (2) For other bacteria, fungi, viruses, and parasites, a minimum of three unique reads were necessary; (3) Pathogens were considered negative if the ratio of microorganism reads per million to the corresponding no-template control was less than 10. In the absence of a universally accepted standard for mNGS interpretation, we utilized a stringent, multi-step validation protocol to define clinical relevance, specifically tailored for the high-risk septic shock population besides bioinformatics thresholds. Specifically, two senior clinicians independently reviewed each candidate pathogen alongside the patient’s clinical presentation, imaging, and concurrent culture results. A result was only recorded as positive if it was deemed the likely causative agent of the septic shock episode, thereby ensuring clinical relevance and minimizing the impact of potential commensal or environmental DNA.

### Statistical analysis

Descriptive statistics were used to summarize the data: categorical variables are presented as frequencies and proportions, while continuous variables are expressed as medians with interquartile ranges (IQR). Continuous variables were compared using the Mann–Whitney *U* test. Categorical variables were assessed using the chi-squared test or Fisher’s exact test. Metagenomic correlation: microbial diversity (alpha and beta diversity) and the abundance of ARGs were compared between the cancer and non-cancer groups. Cox regression models were constructed to identify independent risk factors for ICU and in-hospital mortality, incorporating statistically significant (*p* < 0.05) and clinically relevant variables. Before performing univariate Cox regression analysis, model fitting checks were conducted separately for the tumor group and non-tumor group. For variables with the warning message “Loglik converged before variable 1; coefficient may be infinite”, this indicated the presence of perfect or quasi-perfect separation, where the model failed to stably estimate the hazard ratio (HR). To evaluate whether the prognostic impact of key variables was modulated by malignancy status, we performed formal interaction tests. Interaction terms between cancer vs. non-cancer group and each candidate predictor were incorporated into the Cox proportional hazards models. A *p*-value for interaction (*p*_interaction_) <0.05 was considered to indicate a significant or suggestive differential effect, which provided the statistical rationale for performing stratified multivariable analyses within the cancer and non-cancer cohorts. All statistical analyses and visualization were performed using R software (version 4.0.2) and PRISM 9.0. A two-sided *p*-value <0.05 was considered statistically significant. Heatmaps were generated to visualize the correlation patterns of the studied variables using the pheatmap package in R (version 1.0.13). Rows and columns were hierarchically clustered using average linkage hierarchical clustering with dissimilarity measured by 1 minus Pearson correlation coefficient.

## Result

### Baseline characteristics of the study population

A total of 155 patients with suspected septic shock were screened. After excluding repeat admissions (*n* = 17) and those with incomplete clinical or mNGS data (*n* = 31), 107 patients admitted to the general surgery ICU with septic shock (SSh) were included in this study. The study population comprised 52 cancer patients (48.6%) and 55 non-cancer patients (51.4%). The baseline characteristics of all participants are detailed in Table 1. The median age of the total cohort was 60.00 years (IQR 48.00–70.00), with a slight male predominance (51.4%). Hypertension was the most prevalent comorbidity, affecting 51.4% (*n* = 55) of the patients. The median body mass index (BMI) was 24.49 kg/m^2^ (IQR 22.27–27.34 kg/m^2^). Regarding disease severity at admission, the median APACHE II score was 5.00 (IQR 4.00–7.00), and the median Charlson Comorbidity Index (CCI) was 6.00 (IQR 4.00–10.00).

**Table 1:** Demographic and clinical characteristics of patients with septic shock at ICU admission.

Variable	All patients *n* = 107	Cancer patients *n* = 52	Non-cancer patients *n* = 55	*p*-value
Age (years)	60.00 (48.00–70.00)	68.00 (61.00–73.00)	51.00 (42.00–60.00)	**<0.0001** ^*^
Sex	55 (51.40%)	37 (67.27%)	18 (32.73%)	0.8629
Comorbidities				
Hypertension	55 (51.40%)	37 (32.73%)	18 (71.15%)	**<0.0001** ^*^
BMI	24.49 (22.27–27.34)	23.34 (20.58–25.98)	25.22 (23.57–27.78)	**0.0021** ^*^
Cardiovascular diseases	17 (15.89%)	11 (21.15%)	6 (10.91%)	0.189
Diabetes mellitus	23 (21.50%)	7 (13.46%)	16 (29.09%)	0.0611
Pre-existing renal dysfunction	16 (14.95%)	5 (9.62%)	11 (20.00%)	0.1772
Index				
APACHE II	5.00 (4.00–7.00)	6.00 (4.00–8.00)	5.00 (4.00–6.00)	**0.0019** ^*^
CCI	6.00 (4.00–10.00)	7.00 (4.00–10.00)	4.00 (2.00–8.00)	**0.006** ^*^

Data are expressed in median IQR [25–75] or *n*/*N* (%). Bold values and * indicate *P* < 0.05. BMI, body mass index; CCI, Charlson Comorbidity Index.

### Comparison between cancer and non-cancer patients with SSh

Significant differences in baseline demographics and clinical status were observed between cancer and non-cancer patients (Table 1; Supplementary Table S2). Cancer patients were significantly older than non-cancer patients (median 68.00 years vs. 51.00 years, *p* < 0.0001). Conversely, non-cancer patients exhibited a higher prevalence of hypertension (71.15% vs. 32.73%, *p* < 0.0001) and a higher median BMI (25.22 vs. 23.34 kg/m^2^, *p* = 0.0021). Severity scores were notably higher in the cancer group, including both the APACHE II score (6.00 vs. 5.00, *p* = 0.0019) and the CCI (7.00 vs. 4.00, *p* = 0.006). In terms of clinical and laboratory findings during the ICU stay (Table 2), cancer patients demonstrated a higher neutrophil ratio (87.00% vs. 82.60%, *p* = 0.0088) and lower platelet counts (161.0 vs. 268.0 × 10^9^/L, *p* = 0.0157) compared to non-cancer patients. In the cancer group, the primary malignancies were predominantly located in the gastrointestinal tract and (*n* = 33, 63.46%) hepatobiliary system (*n* = 16, 30.77%) (Supplementary Table S3). Regarding the TNM clinical stage, a significant proportion of patients presented with advanced disease: 20 patients (38.46%) were classified as Stage III and 11 patients (21.15%) as Stage IV, reflecting the high disease burden in this ICU population. 21 patients (40.38%) were at Stage I or II. The majority of patients (*n* = 28, 53.85%) developed sepsis following major surgical resection (within 7 days of ICU admission), highlighting the complex surgical background of oncological sepsis. Active chemotherapy or immunotherapy within the past 30 days was reported in 19 patients (36.39%).

**Table 2:** Comparison of microbiological findings, clinical outcomes, and antimicrobial management between cancer and non-cancer groups.

Variable	All patients *n* = 107	Cancer patients *n* = 52	Non-cancer patients *n* = 55	*p*-value
Type of simples				
Blood	107 (100%)	52 (100%)	55 (100%)	>0.9999
Bile	13 (12.15%)	6 (11.54%)	7 (12.73%)	>0.9999
Ascitic fluid	68 (63.55%)	29 (55.77%)	39 (70.91%)	0.1132
Brochoalveolar lavage fluid	12 (11.21%)	9 (17.31%)	3 (5.45%)	0.0682
Presence of pathogens	75 (70.09%)	28 (53.85%)	47 (85.45%)	**0.0006** ^*^
Bacteremia	38 (35.51%)	13 (25.00%)	25 (45.45%)	**0.0426** ^*^
Presence of resistance genes	59 (55.14%)	25 (48.08%)	34 (61.82%)	0.1767
Laboratory findings				
Creatinine (μmol/L)	65.0 (46.00–99.00)	65.00 (51.00–94.25)	63.00 (40.00–100.00)	0.3012
Hemoglobin (g/dL)	89.00 (80.00–101.00)	86.00 (79.00–94.00)	93.50 (80.00–107.00)	0.0692
WBC count (10^9^/L)	17.9 (13.11–23.62)	17.6 (12.72–20.42)	18.13 (13.83–27.17)	0.1841
Neutrophil ratio (%)	85.00 (77.80–90.50)	87.00 (82.15–92.60)	82.60 (75.90–88.50)	**0.0088** ^*^
Platelet count (10^9^/L)	198.0 (128.0–323.0)	161.0 (122.3–256.8)	268.0 (134.0–357.0)	**0.0157** ^*^
PCT (ng/mL)	1.900 (0.42–9.32)	1.83 (0.48–3.935)	2.900 (0.38–17.61)	0.1095
CRP (mg/L)	167.00 (104.00–211.00)	147.00 (93.25–198.00)	177.0 (104.00–222.00)	0.3122
IL-6 (pg/mL)	175.1 (82.15–416.0)	176.5 (63.34–356.0)	173.9 (84.08–562.9)	0.592
ICU stay and outcome				
Total LOS (days)	21.00 (14.00–34.00)	26.00 (18.25–39.75)	18.00 (13.00–31.00)	0.1039
ICU LOS (days)	10.00 (7.00–18.00)	9.00 (6.00–14.00)	13.00 (7.00–21.00)	**0.0369** ^*^
CRRT	37 (34.58%)	15 (28.85%)	22 (40.00%)	0.3093
Mechanical ventilation	33 (30.84%)	17 (32.69%)	16 (29.09%)	0.8344
Duration of MV (days)	9.00 (5.00–11.00)	7.00 (4.50–13.50)	9.00 (6.00–11.00)	0.6376
ARDS	13 (12.15%)	7 (13.46%)	6 (10.91%)	0.7719
MODS	22 (20.56%)	9 (17.31%)	13 (23.36%)	0.4784
Transfusions	89 (83.18%)	45 (81.82%)	44 (84.62%)	0.7984
Red blood cells	66 (66.36%)	35 (67.31%)	36 (65.45%)	>0.9999
Fresh frozen plasma	85 (79.44%)	44 (80.00%)	41 (78.85%)	>0.9999
Platelet concentrations	32 (29.90%)	13 (23.64%)	19 (36.54%)	0.2048
Duration of antibiotics (days)	8.00 (5.00–16.00)	7.00 (4.25–10.00)	11.00 (5.00–18.00)	**0.0368** ^*^
Mortality in the ICU	12 (11.21%)	5 (9.09%)	7 (13.46%)	0.5491

Data are expressed in median IQR [25–75] or *n*/*N* (%). Bold values and * indicate *P* < 0.05. WBC, white blood cell; PCT, procalcitonin; CRP, C-reactive protein; IL-6, interleukin-6; LOS, length of stay; ARDS, acute respiratory distress syndrome; MODS, multiple organ dysfunction syndrome.

### Metagenomic and microbiological findings

All 107 patients (100%) underwent multi-site sampling for mNGS, including blood, with additional samples obtained from ascitic fluid (63.55%), bile (12.15%), and bronchoalveolar lavage fluid (11.21%). The overall positivity rate for pathogen detection was 70.09% (*n* = 75). Interesting disparities emerged in the infectious profiles between the two groups. Non-cancer patients had a significantly higher rate of positive samples compared to cancer patients (85.45% vs. 53.85%, *p* = 0.0006). Similarly, the incidence of bacteremia was nearly double in the non-cancer group (45.45% vs. 25.00%, *p* = 0.0426). While the presence of resistance genes was numerically higher in non-cancer patients (61.82% vs. 48.08%), this did not reach statistical significance (*p* = 0.1767).

### ICU interventions and clinical outcomes

**Figure 2:**
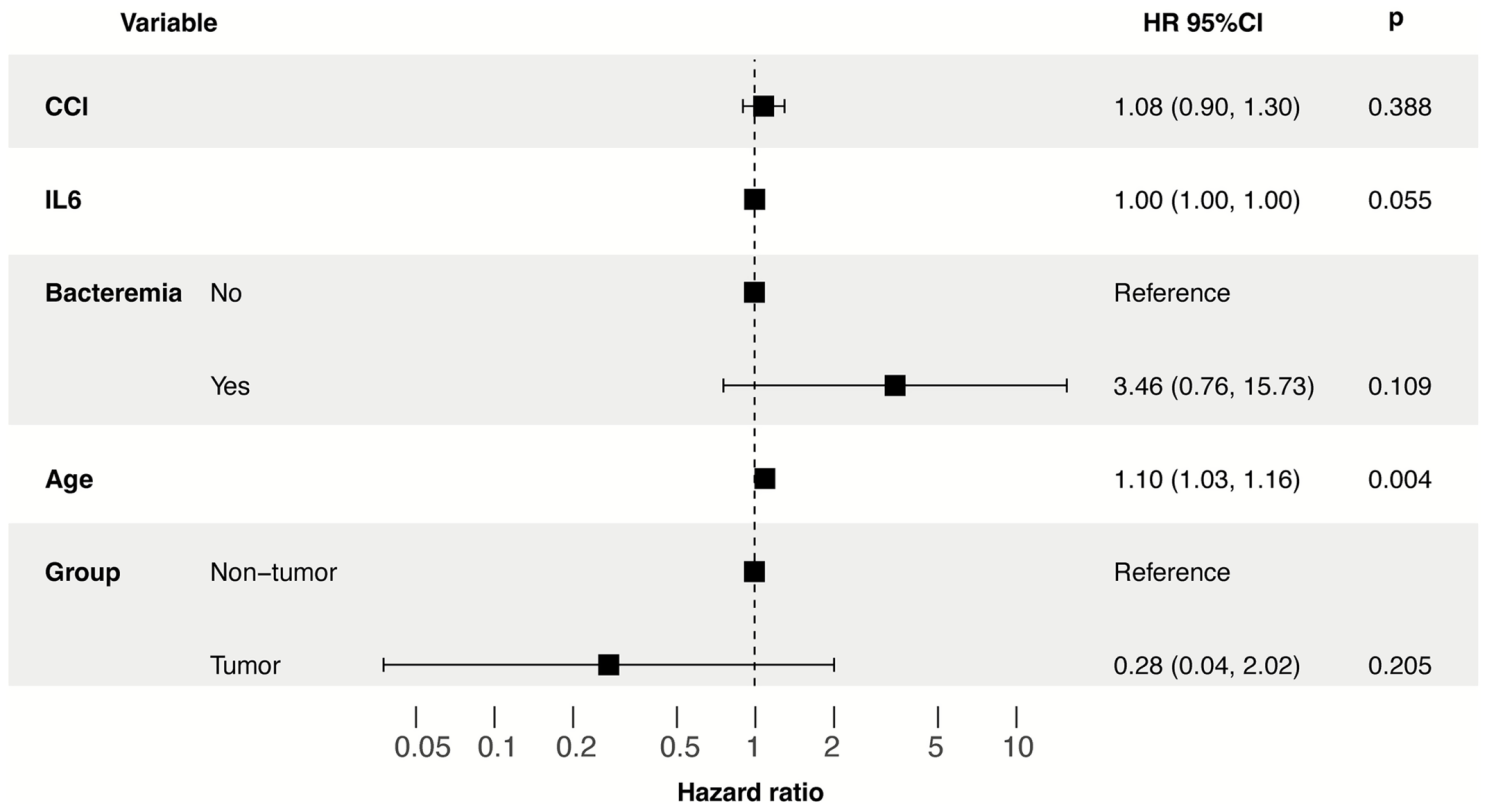
Interaction analysis of prognostic factors in the total cohort. The forest plot displays the Hazard Ratios (HRs) for predictors within the cancer and non-cancer strata derived from a unified Cox model with interaction terms. P interaction values indicate the statistical significance of malignancy as an effect modifier for each variable.

Interventions and outcomes are summarized in Table 2. Regarding respiratory and renal support, 30.84% of patients required mechanical ventilation, and 34.58% received continuous renal replacement therapy (CRRT), with no significant differences between groups. However, the duration of antibiotic therapy was significantly shorter in cancer patients compared to non-cancer patients (7.00 days). To identify independent predictors of prognosis, we performed univariate and multivariate Cox regression analyses for the two cohorts separately, variables were selected based on a predefined criterion (univariable *p* < 0.05 and clinical relevance). In the non-cancer group, univariate analysis revealed that age (HR 1.159, 95% CI 1.043–1.288, *p* = 0.0059) was significantly associated with survival (Table 3). In the multivariate model, age remained a highly significant independent risk factor (HR 1.18, 95% CI 1.02–1.38, *p* = 0.027). In the cancer group, univariate analysis showed that IL-6 levels (HR 1.001, 95% CI 1–1.001, *p* = 0.0109) and WBC count (HR 1.208, 95% CI 1.043–1.400, *p* = 0.0118) were associated with mortality. However, after adjusting for potential confounders in the multivariate analysis, only IL-6 reached statistical significance (HR 1.00, 95% CI 1.00–1.00, *p* = 0.018) (Table 4). To evaluate the modulatory effect of malignancy on prognostic drivers, formal interaction tests were performed between grouping status and candidate predictors. The analysis revealed significant interactions for age (*p*_interaction_ = 0.004), along with a suggestive interaction for the IL-6 (*p*_interaction_ = 0.055) (Figure 2). These results indicate that the prognostic weight of systemic inflammation and baseline physiological reserves differs substantially between the two cohorts, suggesting a trend that IL-6 may have a significant impact on the outcome; however, no statistically significant difference was observed, which may be limited by the small sample size. In contrast, no significant interactions were observed for CCI (*p*_interaction_ = 0.388), bacteremia (*p*_interaction_ = 0.109), suggesting their effects on mortality remained relatively consistent.

**Table 3:** Univariable and multivariable Cox proportional hazards regression analysis of risk factors for mortality in the non-cancer group.

Variable	Univariate analysis		Multivariate analysis	
	HR (95% CI)	*p*-value	HR (95% CI)	*p*-value
CCI	1.209 (0.874–1.671)	0.2512	1.09 (0.56–2.12, *p* = 0.801)	0.801
Age	1.159 (1.043–1.288)	**0.0059** ^*^	1.18 (1.02–1.38, *p* = 0.027)	**0.027** ^*^
IL-6	1.000 (1–1.001)	0.494	1.00 (1.00–1.00, *p* = 0.354)	0.354
BMI	0.844 (0.657–1.083)	0.1828		
Bacteremia	2.465 (0.246–24.692)	0.4427		
Presence of resistance genes	1.31 (0.134–12.767)	0.8161		
CRP	1.001 (0.991–1.011)	0.8913		
PCT	1.026 (0.999–1.053)	0.0556		
WBC	1.023 (0.92–1.139)	0.6709		
CRRT	0.777 (0.101–5.958)	0.8086		
ARDS	5.555 (0.778–39.682)	0.0874		
Time of antibiotics	0.726 (0.497–1.061)	0.0979		
Cardiovascular diseases	3.008 (0.423–21.389)	0.2712		
Hypertension	6.124 (0.673–55.731)	0.1077		
Diabetes mellitus	2.201 (0.333–14.541)	0.4127		
Renal dysfunction	1.735 (0.155–19.366)	0.6545		
Creatinine	1.002 (0.994–1.011)	0.5904		
Neutrophil ratio	1.215 (0.988–1.494)	0.0644		
Hemoglobin	1.001 (0.992–1.009)	0.8445		
Platelet	1.000 (0.993–1.006)	0.8847		

^*Bold values and * indicate *P* < 0.05.^

**Table 4:** Univariable and multivariable Cox proportional hazards regression analysis of risk factors for mortality in the cancer group.

Variable	Univariate analysis		Multivariate analysis	
	HR (95% CI)	*p*-value	HR (95% CI)	*p*-value
CCI	1.039 (0.835–1.292)	0.7319	1.03 (0.82–1.30, *p* = 0.768)	0.768
Age	1.051 (0.964–1.145)	0.2591	1.01 (0.91–1.11, *p* = 0.924)	0.924
IL-6	1.001 (1–1.001)	**0.0109** ^*^	1.00 (1.00–1.00, *p* = 0.018)	**0.018** ^*^
WBC	1.208 (1.043–1.4)	**0.0118** ^*^		
Sex	0.94 (0.15–5.888)	0.947		
BMI	0.899 (0.716–1.129)	0.3595		
Pathogen	0.712 (0.115–4.397)	0.7142		
Bacteremia	5.099 (0.847–30.683)	0.0752		
Presence of resistance genes	3.563 (0.414–30.679)	0.2473		
CRP	1.005 (0.996–1.014)	0.3019		
PCT	1.024 (0.997–1.052)	0.0823		
CRRT	0.281 (0.045–1.747)	0.1733		
ARDS	0.349 (0.037–3.269)	0.3562		
Time of antibiotics	0.937 (0.838–1.048)	0.2567		
Hypertension	1.545 (0.169–14.087)	0.6997		
Diabetes mellitus	0.891 (0.087–9.079)	0.9226		
Renal dysfunction	2.713 (0.443–16.6)	0.2803		
Creatinine	1.011 (0.996–1.025)	0.148		
Neutrophil ratio	1.02 (0.873–1.192)	0.805		
Hemoglobin	1.032 (0.995–1.072)	0.0931		
Platelet	1.000 (0.991–1.008)	0.9393		

Bold values and * indicate *P* < 0.05.

### Metagenomic landscape of pathogens across patient groups

**Figure 3:**
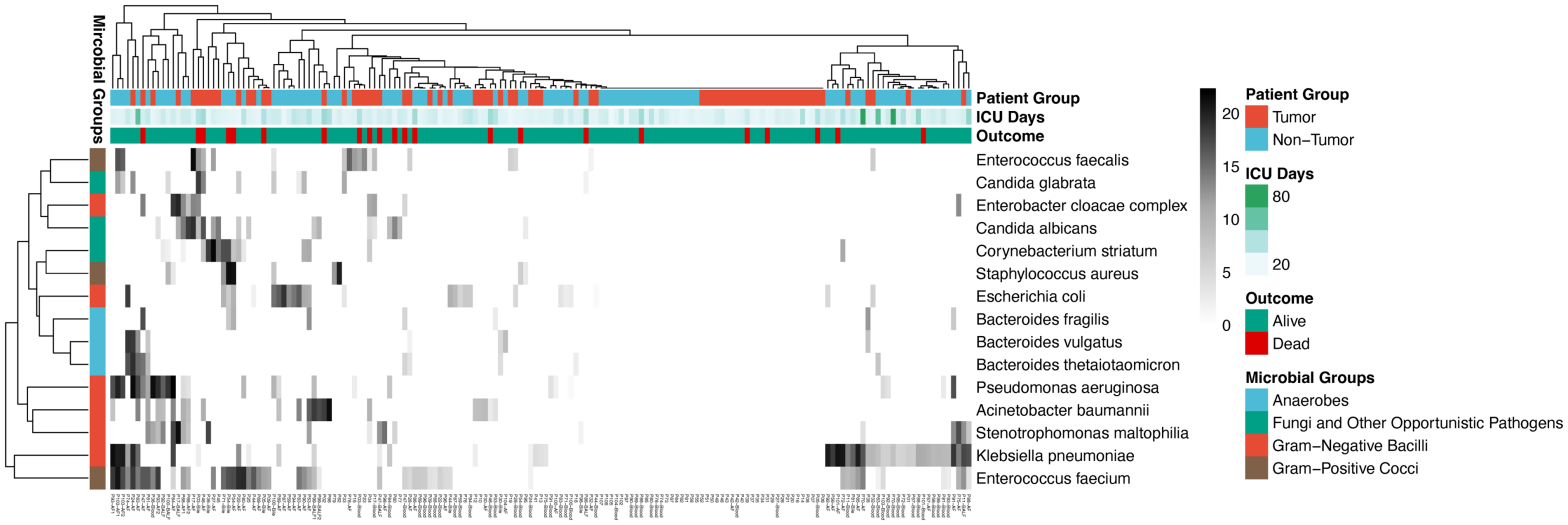
Taxonomic landscape and distribution of pathogens detected by mNGS in surgical ICU patients. The heatmap illustrates the microbial composition identified across multiple clinical specimens, including blood, bile, ascitic fluid, and BALF. Hierarchical clustering indicates the similarity in microbiological profiles among the patients, highlighting the dominant presence of *Klebsiella pneumoniae*, *Escherichia coli*, and *Enterococcus faecium* in the surgical ICU setting.

The microbial landscape, as determined by mNGS across multiple sample types (blood, bile, ascitic fluid, and BALF), is visualized in the hierarchical clustering heatmap (Figure 3 and Supplementary Table S4). Top 15 pathogens were categorized into four major classes: Gram-negative bacilli, Gram-positive cocci, anaerobes, and fungi and other opportunistic pathogens. In term of pathogen distribution, Gram-negative bacilli, including *Klebsiella pneumoniae*, *Escherichia coli*, *Pseudomonas aeruginosa*, and *Acinetobacter baumannii*, were frequently detected across both groups. Gram-positive cocci were primarily represented by *Enterococcus faecium*, *Enterococcus faecalis*, and *Staphylococcus aureus*. Anaerobic species such as *Bacteroides fragilis* and *Bacteroides vulgatus* were notably present, particularly in ascitic fluid samples. Fungal pathogens, specifically *Candida albicans* and *Candida glabrata*, were also identified. The clustering analysis integrates clinical metadata, demonstrating the relationship between the microbial infectome and patient outcomes and ICU duration. While both groups shared common ICU pathogens, the non-tumor group showed a higher overall pathogen positive rate (85.45% vs. 53.85%, *p* = 0.0006) and a higher incidence of bacteremia (45.45% vs. 25.00%, *p* = 0.0426), as detailed in the clinical comparisons.

### Metagenomic profiling of the resistome landscape and virulence factors

**Figure 4:**
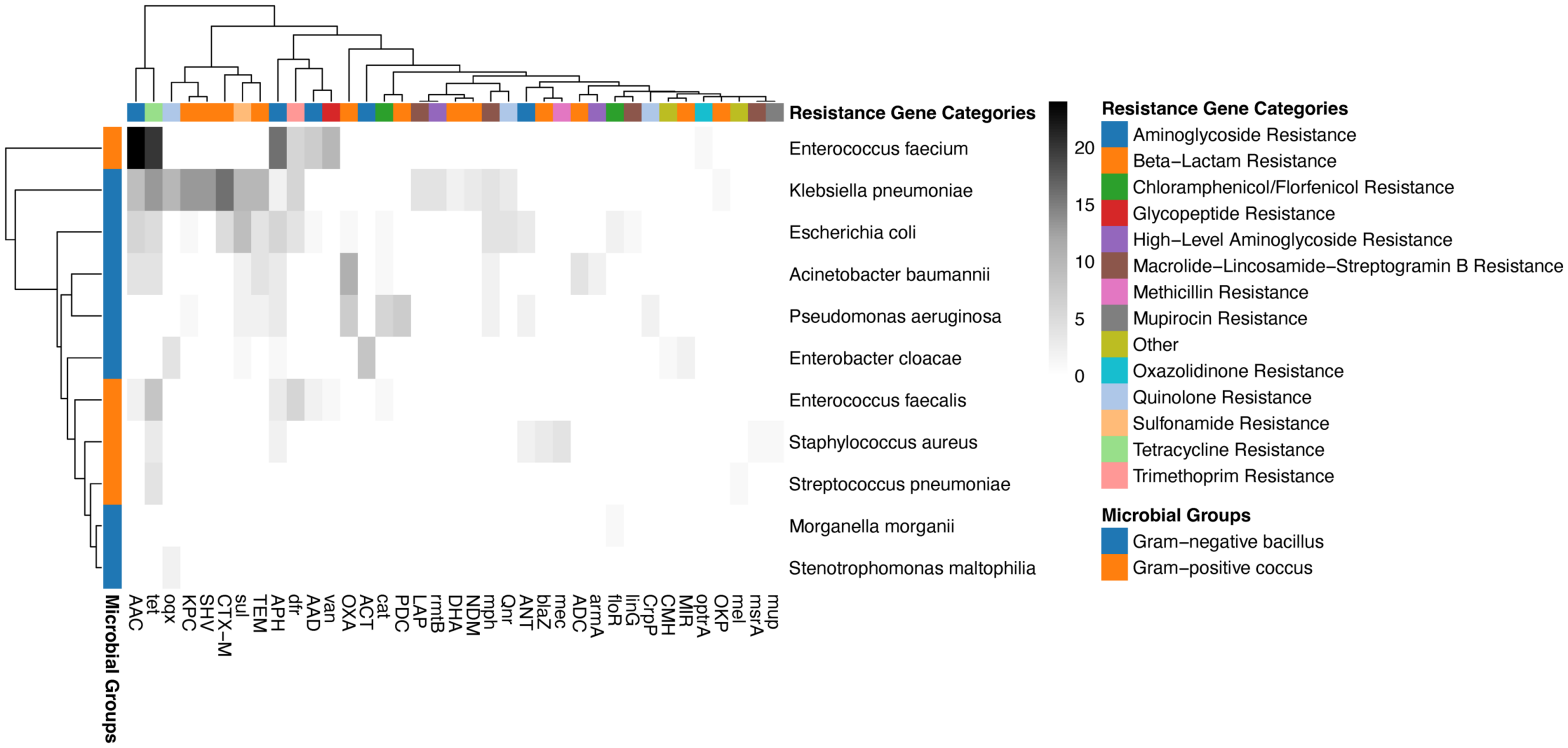
The pathogen-resistome landscape showing the association between microbes and resistance genes. This integrated heatmap visualizes the distribution of ARGs across the identified microbial species. This heatmap reveals the complex multi-drug resistance signatures in patients with septic shock.

To further elucidate the molecular basis of antimicrobial resistance in the General Surgery ICU of Xuanwu Hospital, we integrated taxonomic findings with ARGs annotation using the mNGS data. The resulting pathogen-resistome map, organized by microbial groups and resistance gene categories, provides a high-resolution view of the genetic determinants of resistance (Figure 4 and Supplementary Table S5). The mNGS analysis identified a broad spectrum of ARGs across several major resistance gene groups, including aminoglycoside resistance genes, macrolide-lincosamide-streptogramin resistance, tetracycline resistance, and various beta-lactamases. Specific resistance across different microbials was also found from resistomes. *Klebsiella pneumoniae* and *Escherichia coli* exhibited the most extensive resistome profiles, frequently harboring genes across almost all resistance genes, particularly those conferring resistance to aminoglycosides and beta-lactams. *Acinetobacter baumannii* and *Pseudomonas aeruginosa* also showed targeted resistance patterns, aligning with their clinical roles as multidrug-resistant (MDR) pathogens. In Gram-positive cocci, *Enterococcus faecium* demonstrated a high density of resistance genes, particularly in the macrolide and aminoglycoside groups. *Staphylococcus aureus* was similarly associated with multiple resistance determinants. Although the non-tumor group had a higher overall pathogen detection rate, the pathogen-resistome reveals that once an infection is established, the complexity of the resistome is significant in both groups. This underscores that for cancer patients in the surgical ICU, even a lower microbial load can be associated with highly resistant genetic signatures, complicating empiric therapy. Additionally, we examined the distribution of virulence factors (VFs) and found them in only 6 patients in the cancer group and 2 in the non-cancer group. Most identified VFs were derived from Gram-positive cocci, and all VFs, including iutA, exoT/U/Y, and hysA, are summarized in Table 5.

**Table 5:** List of detected virulence factors from microbials.

Patient	Group	Microbial	Virulence factors
P02	Cancer	*Acinetobacter baumannii*	vgrG/tssI, adeH, csuA/B, ompA, pgaA
P08	Cancer	*Streptococcus pneumoniae*	cbpA/pspC, hysA, lytA, nanA, pavA
P13	Cancer	*Streptococcus pneumoniae*	hysA
P25	Cancer	*Enterococcus faecium*	scm
P44	Cancer	*Enterococcus faecium*	ecbA/fss3, scm
P45	Cancer	*Streptococcus pneumoniae*	cbpA/pspC, pavA, nanA
P82	Non-cancer	*Staphylococcus aureus*	clfB, clfA, fnbB, spa, coa
P93	Non-cancer	*Klebsiella pneumoniae*	iutA
P93	Non-cancer	*Pseudomonas aeruginosa*	iutA, exoT, exoU, exoY, hcp1, lasB

## Discussion

The management of septic shock in surgical ICU patients requires rapid and precise pathogen identification, particularly in the oncological population where immune vulnerability and complex surgical backgrounds coexist (Amanati et al., 2021). Our study provides a high-resolution comparison of the microbial and resistance landscapes between cancer and non-cancer patients, offering critical insights into how molecular diagnostics can be translated into personalized antimicrobial strategies. Sepsis in cancer patients is not merely an infection but a complex interplay between tumor-induced immune dysregulation and the host’s inflammatory response (Mirouse et al., 2020). Our results showed that IL-6 and WBC count were independent predictors of mortality in the cancer group, whereas age dominated in non-cancer patients. Importantly, these associations remained significant after adjusting for baseline imbalances in age and comorbidity burden, suggesting that acute inflammatory dysregulation, rather than baseline fragility alone, drives mortality in surgical oncology patients. This reflects the unique pathophysiological state of “immunological exhaustion” or “hyper-inflammation” characteristic of oncological sepsis.

Cancer patients often present with structural barriers’ disruption due to surgery or radiotherapy, facilitating the translocation of endogenous microbiota (Alexander et al., 2017), and it is reported that gut-derived multidrug-resistant *Klebsiella pneumoniae* colonization in pancreatic cancer tissues (Zhao et al., 2025). Furthermore, the baseline immunosuppression, compounded by chemotherapy-induced neutropenia, alters the sepsis phenotype (Rashidi et al., 2020). In these patients, the magnitude of the cytokine storm often exceeds the compensatory anti-inflammatory response, leading to rapid multi-organ failure (Knight et al., 2017). This highlights that for cancer patients, targeting the microbial trigger must be synchronized with modulating the dysregulated immune response to improve prognosis.

In the golden hour of septic shock management, every hour of delay in appropriate antibiotic administration increases mortality by approximately 7.6% (Gaieski et al., 2010). For cancer patients, who possess lower physiological reserves, this window is even narrower. Traditional culture-based methods, with a turnaround time of 48–72 h and low sensitivity for fastidious or prior-antibiotic-exposed organisms, often fall short (Pinzauti et al., 2025). In our cohort, the use of mNGS was associated with shorter antibiotic durations in cancer patients despite their higher severity scores. While the retrospective nature of this study precludes a direct causal link, these observations suggest that the rapid identification of microbial targets may support earlier transition from empiric broad-spectrum therapy to targeted strategies. By providing results within 24 h, mNGS allows clinicians to transition from empiric broad-spectrum therapy to targeted antimicrobial strategies significantly faster than traditional methods. This timeliness is a decisive factor in preventing antibiotic-associated toxicities and *Clostridioides difficile* infections, which are particularly devastating in oncological surgical patients (Yepez Guevara et al., 2021).

The integration of taxonomic data with ARGs provides a powerful tool for risk-stratified intervention. We identified high-density resistance clusters in *Klebsiella pneumoniae* and *Enterococcus faecium*, harboring genes across beta-lactamases (*blaKPC*, *blaNDM*), aminoglycoside resistance (*armA*, *aac*), and macrolides. The benefit of mNGS-based resistome profiling for cancer patients is twofold. First, it identifies high-risk resistotypes. For example, the detection of carbapenemase genes provides molecular evidence that could potentially inform the use of novel agents like ceftazidime/avibactam or aztreonam, rather than waiting for phenotypic sensitivity (Hidalgo-Tenorio et al., 2024). This pre-emptive precision can be life-saving. Second, it informs microbiome-sparing therapy. By identifying the specific ARGs present, clinicians can avoid non-targeted therapy with ultra-broad-spectrum carbapenems if the detected pathogen is susceptible to narrower agents. Preserving the gut microbiome is increasingly recognized as critical in oncology, as a healthy microbiome modulates the host’s response to immunotherapy and chemotherapy (Lei et al., 2025). Therefore, the mNGS-driven resistome assessment does not just target the pathogen; it protects the patient.

Additionally, our research utilized multi-site sampling, including blood, bile, ascitic fluid, and BALF. The heatmap revealed that pathogens are often compartmentalized. For surgical oncology patients, the primary site of infection may harbor the true “culprit” pathogen, while blood mNGS remains negative or shows only secondary translocation. By analyzing the infectome across multiple compartments, we observed that *Enterococcus faecium* and *Klebsiella pneumoniae* often co-exist in ascitic fluid and bile, suggesting a polymicrobial synergy that drives septic shock in abdominal surgery. The ability of mNGS to unravel this polymicrobial landscape, which is frequently masked by the overgrowth of a single species in traditional cultures, allows for a more balanced and effective antibiotic strategy that covers all relevant pathogenic components. Furthermore, although we recorded the use of life-support interventions such as CRRT and mechanical ventilation, we did not perform a granular analysis of their specific interactions with the microbial resistome. Given that sampling occurred early in the ICU course, these interventions primarily reflect the severity of organ dysfunction rather than prolonged environmental pressure.

Additionally, we found several VFs that may help understand sepsis in cancer patients, despite their rather low detection rate. The detection of potent toxins and enzymes like lasB, Hysa and lyta suggests a mechanism of accelerated tissue degradation. In cancer patients, where anatomical barriers are already compromised by surgical trauma or tumor invasion, these enzymes can facilitate the systemic dissemination of pathogens (Saint-Criq et al., 2018). pgaA in *Acinetobacter baumannii* and fnbB in *Staphylococcus aureu* underscores the challenge of managing biofilm-associated infections (Wang et al., 2023). These factors facilitate the establishment of bacterial adhesion to biliary stents and abdominal drains. As for exoT/U/Y, a potent phospholipase, is known to cause rapid cell membrane lysis and lung injury, while lasB degrades host structural proteins and immune components (Deruelle et al., 2025). The results found the prevalence of iron acquisition systems (iutA, adeH) and immune evasion factors (spa, ompA) is particularly alarming for the cancer population. In the low-iron environment of the blood and bile, pathogens carrying these siderophores gain a decisive competitive advantage (Chan and Lewis, 2022). Furthermore, factors like spa (staphylococcal protein A) capsule regulator allow pathogens to escape from the host’s already weakened neutrophils and macrophages (Balachandran et al., 2018). The characterization of these VFs opens new avenues for precision therapy beyond traditional antibiotics. LasB inhibitors or siderophore-quencing agents could theoretically be used as adjunctive therapies to disarm pathogens without exerting the same selective pressure as bactericidal drugs. Additionally, targeting biofilm-associated proteins like pgaA might enhance the efficacy of stent-based treatments. In the era of increasing multidrug resistance, focusing on these virulence-associated pathogenic mechanisms may offer a more viable strategy for improving the prognosis of immunosuppressed oncology patients with septic shock.

Despite the promising findings, this study has limitations. As a retrospective, monocentric study, the sample size, particularly the number of mortality events in the cancer group, was relatively small, which may have limited the power of the multivariable Cox analysis. Also, patients who died within 24 h of ICU admission were excluded, potentially introducing a survivor bias toward those stable enough to undergo comprehensive metagenomic testing. Thus, we cannot conclude that mNGS-guided interventions directly improved clinical outcomes. The observed shorter antibiotic duration may be influenced by unmeasured antimicrobial stewardship protocols or clinician preference, rather than mNGS results alone. Additionally, mNGS cannot currently distinguish between DNA from live bacteria and DNA from degraded organisms (Chiu and Miller, 2019). While sampling was standardized within 24 h of septic shock, indication bias remains a concern; sicker patients may have been prioritized for multi-site sampling. Furthermore, this study was conducted in a single-center surgical ICU focusing on gastrointestinal and hepatobiliary cancers, which limits the generalizability of our findings to non-surgical settings, other cancer types, or different healthcare systems. However, in the clinical context of septic shock, the presence of high-abundance pathogen DNA in a sterile site is almost always clinically significant (Sinha et al., 2025). Future prospective studies should focus on integrating mNGS with host-response transcriptomics. By combining pathogen detection with host RNA signatures, we can better differentiate between active infection and sterile inflammation, further refining the specific antimicrobial strategies needed to improve the prognosis of cancer patients.

## Conclusion

In conclusion, septic shock in cancer patients exhibits distinct microbial and resistance signatures compared to non-cancer populations. Using multi-site mNGS, we identified *Klebsiella pneumoniae* and *Enterococcus faecium* as key pathogens harboring complex resistance genes. For cancer patients, prognosis is closely tied to inflammatory intensity and specific resistance determinants rather than age. By mapping the pathogen-resistome landscape, mNGS enables targeted antimicrobial strategies essential for therapeutic precision. These findings support molecular-based management to enhance survival for patients undergoing cancer therapy in the ICU.

## Data Availability

The original contributions presented in the study are publicly available. The raw metagenomic datasets can be found in the GSA repository, and Accession Number CRA039179.This data can be found here: https://ngdc.cncb.ac.cn/gsa/browse/CRA039179.
